# The Invasion and Encapsulation of the Entomopathogenic Nematode, *Steinernema abbasi,* in *Aedes albopictus* (Diptera: Culicidae) Larvae

**DOI:** 10.3390/insects11120832

**Published:** 2020-11-26

**Authors:** Wei-Ting Liu, Tien-Lai Chen, Roger F. Hou, Cheng-Chen Chen, Wu-Chun Tu

**Affiliations:** 1 Department of Entomology, National Chung Hsing University, Taichung 402, Taiwan; wtliu@dragon.nchu.edu.tw (W.-T.L.); daycome@alumni.nchu.edu.tw (T.-L.C.); rhou@dragon.nchu.edu.tw (R.F.H.); 2 Department of Tropical Medicine, National Yang-Ming University, Taipei 112, Taiwan; mosquito@ym.edu.tw

**Keywords:** *Aedes albopictus*, *Steinernema abbasi*, defense reaction, encapsulation

## Abstract

**Simple Summary:**

The Asian tiger mosquito, *Aedes albopictus*, is widely considered to be one of the most dangerous vectors transmitting human diseases. Meanwhile, entomopathogenic nematodes (EPNs) have been applied for controlling insect pests of agricultural and public health importance for years. However, infection of mosquitoes with the infective juveniles (IJs) of terrestrial-living EPNs when released to aquatic habitats needs further investigated. In this study, we observed that a Taiwanese isolate of EPN, *Steinernema abbasi*, could invade through oral route and then puncture the wall of gastric caecum to enter the body cavity of *Ae. albopictus* larvae. The nematode could complete infection of mosquitoes by inserting directly into trumpet, the intersegmental membrane of the cuticle, and the basement of the paddle of pupae. After inoculation of mosquito larvae with nematode suspensions, the invading IJs in the hemocoel were melanized and encapsulated and only a few larvae were able to survive to adult emergence. The mosquitocidal effect of *S. abbasi* could be due to its overload or destruction of the mosquito defense systems. Eventually, the mosquito larvae failed to recover and died a few days after infection. Our results suggest that the terrestrial EPN, *S. abbasi*, is effective against the aquatic living mosquitoes.

**Abstract:**

The Asian tiger mosquito, *Aedes albopictus*, is of crucial concern to the public and veterinary health because of its vector role in transmission of several mosquito-borne diseases. Over the past decades, entomopathogenic nematodes (EPNs) have been used to control important agricultural insect pests and are considered to be effective against mosquitoes as well. The objectives of this study were to investigate the mosquitocidal effects of *Steinernema abbasi* to *Ae. albopictus* and the encapsulation processes of invading nematodes in the mosquito host. In this study, we found that *S. abbasi* was pathogenic to 3rd and 4th instar larvae of *Ae. albopictus* by entering the hemocoel of the 3rd and 4th instar larvae mainly through mouth and gastric caecum or by penetrating pupae through the intersegmental membrane or trumpet. The mosquito larvae infected with a single nematode caused a high mortality. Although EPNs in the hemocoel of mosquitoes were melanized and encapsulated, most *Ae. albopictus* larvae failed to survive after infection with *S. abbasi*. Overall, we demonstrated that *S. abbasi* is pathogenic to *Ae. albopictus* larvae, suggesting that this *S. abbasi* isolate has potential as a biocontrol agent for managing this vector mosquito.

## 1. Introduction

In the past few decades, the emergence and re-emergence of several vector-borne viral diseases have threatened human health [1,2]. The Asian tiger mosquito, *Aedes albopictus*, is a daytime biting mosquito [3] and is known to transmit several arboviruses including dengue virus, chikungunya viruses, West Nile virus, eastern equine encephalitis virus, and Japanese encephalitis virus in nature [4,5,6,7]. In addition, *Ae. albopictus* is also an important vector of dog heartworm parasites, *Dirofilaria immitis* and *D. repens* [8]. To date, there is no effective therapeutics or vaccine available for the treatment of these mosquito-borne diseases. Conventional chemical insecticides are commonly applied to suppress the epidemics of mosquito-transmitted diseases [9,10,11]. Nevertheless, there has been progressive development of insecticide resistance in the vector populations over the years [12]. Inherited resistance to chemical insecticides in mosquitoes usually arises through one of two mechanisms, i.e., target-site resistance mutations and/or metabolic-based resistance [13]. Moreover, application of chemical insecticides could cause hazard to non-target organisms, contaminate the surrounding environment, and bioaccumulate through biomagnified food chain [14]. Biological control is thus considered as an environmentally friendly alternative to chemical insecticides.

Since 1980s, the entomopathogenic nematodes (EPNs) have been reported to be effective biocontrol agents for managing a diverse number of the insect pests of crops including those on foliar, soil surface and cryptic or subterranean habitats [15,16,17,18,19]. Recently, EPN-based products have already been available in many countries [20,21]. The majority of EPNs belong to two genera *Steinernema* and *Heterorhabditis*, which can kill insects rapidly by symbiotic bacteria of the genera, *Xenorhabdus* and *Photorhabdus*, respectively [22,23]. In the process of infection, the infective juveniles (IJs) of EPNs penetrate through natural body openings of insects such as mouth, spiracles, and anus [24], while some heterorhabditids can also penetrate through the intersegmental membranes of the insect using their dorsal tooth [25,26,27]. After penetration, the symbiotic bacteria are released from the nematode gut causing septicemia [28] and the death of insects within approximately 48 h [17,29].

Since EPNs are able to infect, develop, and complete their life cycle in about 250 insect species belonging to 75 families of 11 orders [30,31], they can be a potential biological control agent against insect pests. Importantly, EPNs can be easily mass-produced and applied using the available spray equipment and being safe to the environment [23,30]. For management strategies of *Ae. albopictus*, it is necessary to know the breeding habitats of *Aedes* species. Their females usually prefer to oviposit either in areas of relatively clean water or small containers placed in home yards such as uncovered barrels, plastic buckets, water storage drums, jars, discarded tires, flower vases, and trash cans [32,33,34,35]. Although both steinernematids and heterorhabditids are terrestrial nematodes, the aquatic habitat is also best for the nematode survival [36,37,38,39,40,41]. In aquatic habitat, IJs of terrestrial nematodes sink to the bottom of the containers where mosquito larvae frequently feed on the organic detritus and may swim actively in search of hosts resulting in successful infection of mosquitoes [40,42]. EPNs can infect a wide range of susceptible mosquito species [36,37,39,40,41,43]. Therefore, we investigated the mosquitocidal effects of *S. abbasi*, which is an indigenous nematode isolated from Taiwan by Liao et al. (2001) [44], against *Ae. albopictus* in aquatic habitats, and observations on routes of entry of *S. abbasi* into *Ae. albopictus*, and encapsulation of the invading nematodes in the host mosquitoes.

## 2. Materials and Methods

### 2.1. Mosquito Rearing

Colonies of *Ae. albopictus* were originally collected from Kaoshiung, Taiwan, in 1998. This mosquito strain has been maintained in the laboratory since then. Mosquitoes were reared at 28 ± 1 °C and 80% ± 10% relative humidity (RH) on a photoperiod of 12:12 h (L:D) under standard laboratory conditions. Adults were provided with a 10% sugar solution on soaked cotton rolls, and females were blood-fed biweekly using anaesthetized mice. Larvae were fed daily on a 1:1 mixture of goose liver powder and yeast powder.

All mice that were used frequently as a source of blood for mosquitoes were treated in accordance with the institutional Animal Care and Use Committee (IACUC) of NCHU, Taiwan. The study protocols were reviewed and approved by the Committee on Animal Research and Care in NCHU (No. 102-76, 23 October 2013 to 17 October 2018). All efforts were made to minimize suffering of mice.

### 2.2. Nematode Propagation

Two *Steinernema* nematodes were studied in the laboratory: *S. abbasi* was the stock colony maintained at Insect Pathology Laboratory of our department, whereas *S. carpocapsae* (Weiser; All strain) was obtained from BioSys Inc. (Columbia, MD, USA) given by Frank F. N. Chang, Temple University, USA in 1990. Nematode propagation was conducted by the methods described by Liao et al. (2001) [44]. Both nematodes were produced by passing through larvae of *Spodoptera litura* (Fabricius). The IJs were collected with the White trap and then were surface sterilized three times with 0.1% formalin solution. After sterilization, the nematodes were washed three times with sterilized distilled water. The IJs were transferred to a 5.5-cm petri dish containing a sponge pieces soaked with sterilized distilled water. Cultures of *S. carpocapsae* was maintained at 26 ± 2 °C and 90% ± 10% RH, while *S. abbasi* was reared at 20 ± 1 °C and 90% ± 10% RH in the dark.

### 2.3. Infection Assay of Ae. albopictus

The infection assay was carried out as described by Beresky and Hall (1977) [45] with some modifications. Briefly, each group of thirty 1st, 2nd, 3rd, or 4th instar larvae or pupae of *Ae. albopictus* was rinsed in 30 mL of sterilized water and then placed in a 250-mL glass beaker (6 cm in diameter) with a water level of ca. 1.5 cm from the bottom. Five concentrations of nematodes were tested, i.e., 1, 10, 100, 1000, and 10,000 IJs/mL. Distilled water was used for controls. Mortality rates of *Ae. albopictus* were recorded every 4 h for 72 h after inoculation, and were compared using Tukey’s honestly significant difference (HSD) test. A probit analysis was performed to calculate the lethal time values (LT_50_ and LT_90_) of *Ae. albopictus*. *Steinernema carpocapsae* could reduce larval density and adult emergence of *Aedes* spp. [46] and is also a commercialized entomopathogenic nematode. We thus used it as a control for comparing the infectivity of *S. abbasi* to *Ae. albopictus* in this study. There were four replicates with each treatment containing thirty larvae or pupae of *Ae. albopictus* in graded concentrations of nematode.

### 2.4. Observations on Routes of Entry of S. abbasi Into Ae. albopictus

Optical microscopy was performed for observations on entry of EPNs into mosquitoes. The 4th instar larvae of *Ae. albopictus* were rinsed in 30 mL of sterilized water (1 × 10^4^ IJs/mL) and placed in a glass beaker for 30 min. After the incubation period, the mosquitoes were washed three times with sterilized distilled water, and immediately the entire intestinal tract of the mosquito was dissected and photographed under a microscope (MZ75; Leica microsystems, Wetzlar, Hesse, Germany) in cold phosphate buffered saline (150 mM NaCl, 1 mM CaCl_2_, 2 mM KCl, and 1 mM NaHCO_3_, pH 7.0; Merck KGaA, Darmstadt, Hesse, Germany).

For entry through cuticle or natural body openings of larvae and pupae, fifty larvae or pupae were placed on filter paper and were incubated at 0.5 mL of sterilized water (1 × 10^3^ IJs/mL) for 30 min. After the incubation period, the larvae or pupae were fixed with 2.5% glutaraldehyde (Merck KGaA, Darmstadt, Hesse, Germany) in 0.1 M sodium cacodylate buffer (pH 7.4; Merck KGaA, Darmstadt, Hesse, Germany) for 3 h at 4 °C. The fixed samples were washed three times in 0.33 M cold sucrose (Merck KGaA, Darmstadt, Hesse, Germany) in 0.1 M sodium cacodylate buffer for 15 min each, and were post-fixed in 1% osmium tetroxide (Acros Organics, Morris Plains, NJ, USA) in 0.1 M sodium cacodylate buffer (pH 7.4) for 1 h at 4 °C. Subsequently, the fixed samples were washed three times in sterilized distilled water for 15 min followed by dehydration in graded acetone series (50–100%), were critical point-dried using a critical-point dryer (Hitachi, Tokyo, Japan), and then visualized and photographed using a stereomicroscope (Olympus, Tokyo, Japan).

### 2.5. Observations on Encapsulation of Invading Nematodes in Ae. albopictus

Encapsulation of invading *S. abbasi* was observed using an optical microscopy as described above. The number of the penetrating nematodes and the encapsulated nematodes were counted and photographed under a microscope (MZ75; Leica microsystems, Wetzlar, Hesse, Germany).

Transmission electron microscopy (TEM) was performed for ultrastructural observations on encapsulation of invading *S. abbasi*. In brief, at 10 min, 30 min, 1 h, 2 h, 24 h, or 48 h after inoculation, body fragments of the EPN-infected 4th instar larvae were excised and fixed with 2.5% glutaraldehyde (Merck KGaA, Darmstadt, Hesse, Germany) in 0.1 M sodium cacodylate buffer (pH 7.4; Merck KGaA, Darmstadt, Hesse, Germany) for 12 h at 4 °C. The fixed samples were washed three times in 0.33 M cold sucrose (Merck KGaA, Darmstadt, Hesse, Germany) in 0.1 M sodium cacodylate buffer for 15 min each, and were post-fixed in 1% osmium tetroxide (Acros Organics, Morris Plains, NJ, USA) in 0.1 M sodium cacodylate buffer (pH 7.4) for 2 h at 4 °C. The resulting samples were washed three times in sterilized distilled water for 15 min each, dehydrated in a series of ethanol solutions, and then embedded in Epon 812 resin (Electron Microscopy Science, Hatfield, PA, USA). The blocks were sectioned using an ultramicrotome (Ultracut; Leica Microsystems, Wetzlar, Hesse, Germany). The ultrathin sections were placed on formvar-coated copper grids, post-stained with 2.5% uranyl acetate (Merck KGaA, Darmstadt, Hesse, Germany) and 1% lead citrate (Merck KGaA, Darmstadt, Hesse, Germany), and then visualized and photographed using a transmission electron microscope (JEM 2000EX II; Japanese Electron Optic Laboratory, Tokyo, Japan).

## 3. Results

### 3.1. Virulence of S. abbasi and S. carpocapsae to Ae. albopictus Larvae

*S. carpocapsae* is a commercial product of EPNs and is one of the most commonly applied species for controlling a variety of insects in agricultural pest control [15]. Therefore, we selected *S. carpocapsae* for comparing the mosquitocidal effect of *S. abbasi*. We first examined mosquitocidal effects of *S. abbasi* isolated from Taiwan on each developmental stage of mosquito. At 72 h after inoculation with *S. abbasi* and *S. carpocapsae*, no mortalities of the 1st and 2nd instar larvae and pupae of *Ae. albopictus* were observed, while those of the 3rd and 4th instar larvae were increased as elevated EPN concentrations (Figure S1). Subsequently, both LT_50_ and LT_90_ values were determined with treatments of 30 mosquito larvae. Most of LT_50_ and LT_90_ values of *S. abbasi* to *Ae. albopictus* were similar and no significant difference at a concentration of 1 × 10^4^ IJs/mL although slightly lower than those of *S. carpocapsae*. However, at a concentration of 1 × 10^3^ IJs/mL, LT_50_ and LT_90_ values of *S. abbasi* to 3rd instar larvae of *Ae. albopictus* was significantly lower than those of *S. carpocapsae*. In addition, comparing with the development time of larval stages of untreated larvae, the next molt and pupation of *Ae. albopictus* larvae seem to be prolonged after inoculation (Table 1 and Table S1). The mortality rate did not reach 90% within 72 h at a concentration of 1 × 10^3^ IJs/mL (Table 1). Collectively, the inoculation of *S. abbasi* caused effects similar to *S. carpocapsae* infection.

### 3.2. Routes of Entry of S. abbasi Into Ae. albopictus

**Figure 1:**
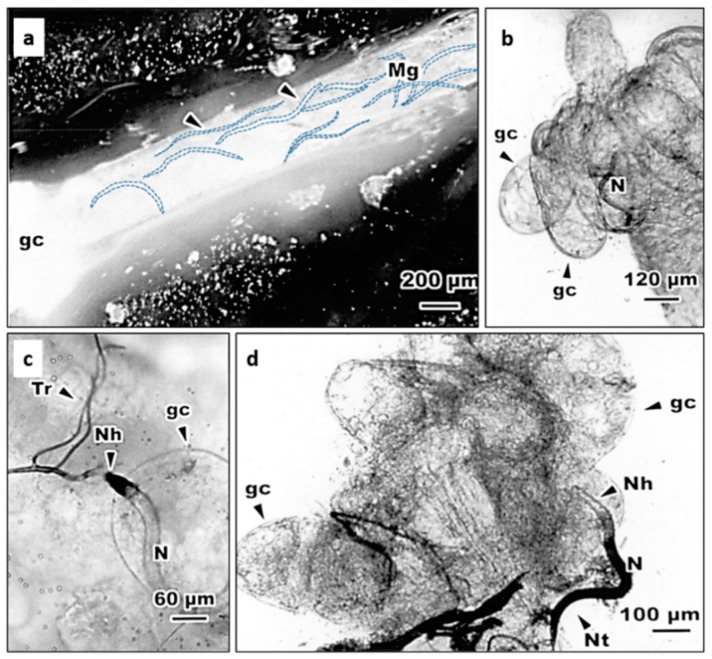
The penetration of *S. abbasi* to the hemocoel of the 4th instar larva of *Ae. albopictus* at 10 min after inoculation. (**a**) Nematode in the midgut of the 4th instar larva of *Ae. albopictus*. gc: gastric caecum, Mg: midgut. Arrow head: *S. abbasi*. (**b**) Nematode in the gastric caecum of the 4th instar larva of *Ae. albopictus*. (**c**) Nematode penetrated by its head from gastric caecum to the hemocoel of the 4th instar larva of *Ae. albopictus*. (**d**) Nematode penetrated by its tail from gastric caecum to the hemocoel of the 4th instar larva of *Ae. albopictus*. N: *S. abbasi*, Nh: nematode head, Nt: nematode tail, gc: gastric caecum, Tr: tracheae.

**Figure 2:**
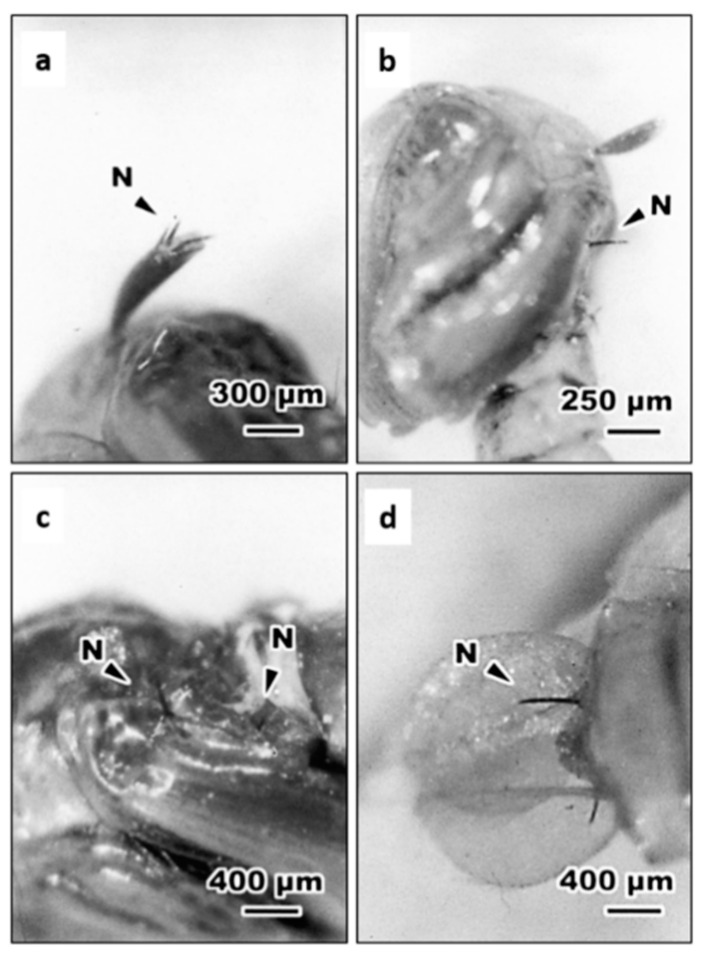
The penetration of *S. abbasi* into the pupa of *Ae. albopictus*. (**a**) Nematode penetrated through the trumpet of *Ae. albopictus* pupa. (**b**) Penetrated through the intersegmental membrane of the cephalothorax of *Ae. albopictus* pupa. (**c**) Penetrated through the intersegmental membrane of the abdomen of *Ae. albopictus* pupa. (**d**) Penetrated through the basement membrane of paddle of of *Ae. albopictus* pupa. N = *S. abbasi*.

It was indicated that *Steinernema* nematodes are only able to enter their insect body via natural openings [25,27]. In this study, we further examined histologically the entry of *S. abbasi* into mosquitoes after incubation. Optical microscopic observations showed that, at 10 min after inoculation, IJs of *S. abbasi* aggregated within the midgut of 4th instar larva (Figure 1a). Some IJs were also found in the gastric caecum (Figure 1b) and penetrated through the gastric caecum into the hemocoel (Figure 1c). As penetrated into the hemocoel, IJs within the hemocoel were found melanized, while those remaining in the lumen of gastric caecum were non-melanized (Figure 2c,d). We also observed that the IJs inserted parts of their body into trumpet (Figure 2a), the intersegmental membrane of the cuticle and the basement of the paddle of pupae (Figure 2b–d).

### 3.3. The Encapsulation of S. abbasi in the 4th Instar Larvae of Ae. albopictus

In order to find out whether the nematodes are able to survive within a mosquito larva and to utilize mosquito hemocoel for the mass production of *S. abbasi*, and additionally, whether this *S. abbasi* isolate is capable of producing massive offspring in mosquitoes as a synergistic control agent. The larvae were dissected at several time intervals after inoculation with nematodes, we observed that the invading nematodes were encapsulated after entering the hemocoel of mosquito larvae, indicating that the nematodes are unable to use mosquito body for propagation. Additionally, invasions by a large number of nematodes may overburden the immune system of mosquito larva. Observations from dissecting larvae infected with EPNs showed that as many as 31 melanized nematodes were found in one *S. abbasi*-infected larva, in contrast, the maximal number of melanized EPNs, was only 15 found in one *S. carpocapsae*-infected larva (Table 2 and Table S2). The melanotic capsules were distributed mainly in thorax (76%), followed by the abdomen (20%), and few in head (4%) in *S. abbasi*-infected *Ae. albopictus*, while there were 86% in the thorax, 9% in the abdomen and only 5% in the head in *S. carpocapsae*-infected larvae.

**Figure 3:**
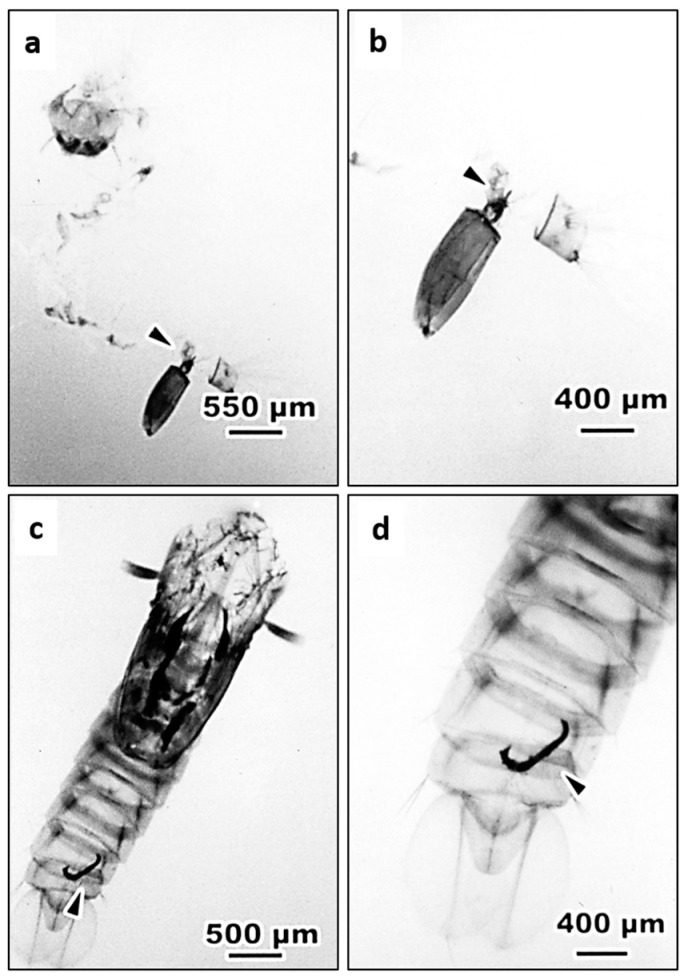
The encapsulated *S. abbasi* extruded from larva or pupa of *Ae. albopictus*. (**a**) The capsule (arrow head) excluded from the end of exuvia in larval abdomen of *Ae. albopictus*. (**b**) The higher magnification of (**a**). (**c**) The capsule (arrow head) extruded from the end of the exuvia in pupal abdomen of *Ae. albopictus*. (**d**) The higher magnification of (**c**).

Among 183 of 4th instar larvae inoculated with *S. abbasi*, only 18 of them were able to survive, pupate, and emerge to adults in which 11 larvae harbored melanized EPNs in their body including 6 with one capsule, 4 with two capsules and 1 with four capsules. Some melanized capsules were extruded from the end of exuvia in larval abdomen (Figure 3a,b) or pupal abdomen (Figure 3c,d). Similarly, in 177 larvae inoculated with *S. carpocapsae*, only 6 of them developed to an adult including 4 without a capsule and 2 with two capsules (Table S2).

### 3.4. The Processes of Encapsulation of S. abbasi in Ae. albopictus

**Figure 4:**
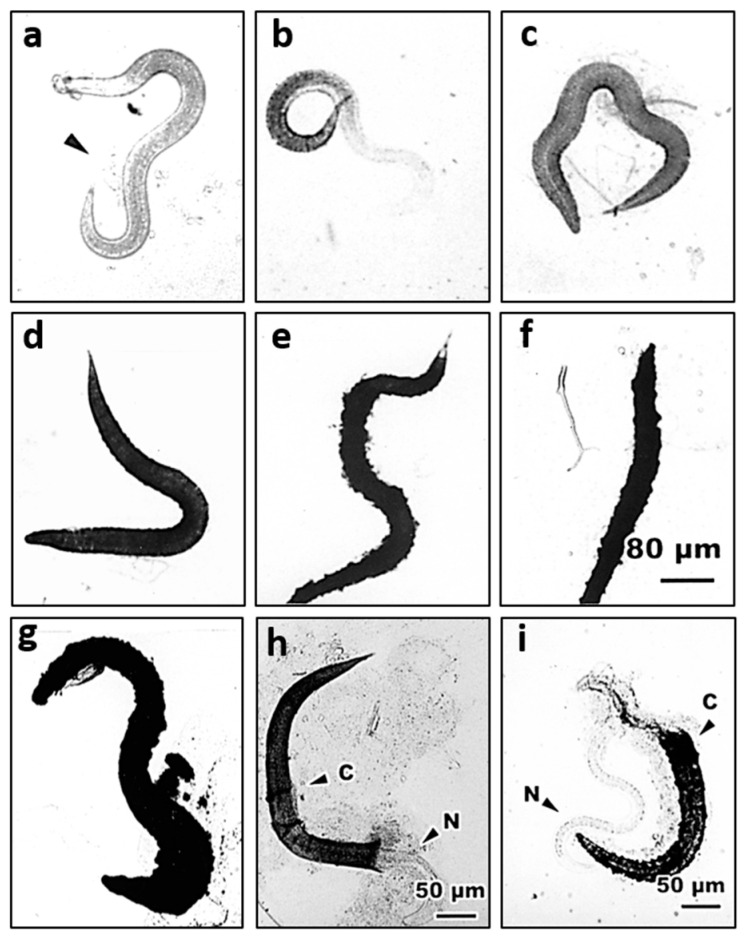
Encapsulation of *S. abbasi* in the hemocoel of 4th instar *Ae. albopictus* larvae. (**a**) At 5 min after inoculation, transparent granules (arrow head) deposited on the surface. (**b**) At 10 min after inoculation, partially encapsulated and slightly melanized. (**c**) At 30 min after inoculation, completely encapsulated and slightly melanized. (**d**) At 1 h after inoculation, melanized and completely covered by capsule materials. (**e**) At 2 h after inoculation, the surface of capsule fiberized. (**f**) At 4 h after inoculation, *S. abbasi* covering with roughly melanized capsule. (**g**) At 8 h after inoculation, *S. abbasi* and the capsules heavily melanized. (**h**) *S. abbasi* (N) partially covered by capsule materials (C). (**i**) An unencapsulated nematode (N) forming an empty capsule (C).

The above results showed that although *S. abbasi* IJs infection caused high mortality of *Ae. albopictus* larvae, they were encapsulated effectively suppressing their development after entering the hemocoel of mosquito larvae (Table 2). Further studies are necessary to clarify whether the encapsulation process or internal structures interfere with the nematode infection. Optical micrographs showed that transparent granules were found to be deposited on the surface of *S. abbasi* at 5 min after inoculation (Figure 4a). Subsequently, the transparent capsule was slightly melanized at 10 min after inoculation (Figure 4b). The invading nematode was completely encapsulated and was slightly melanized showing a smooth surface after 30 min (Figure 4c). The transparent capsule enclosing *S. abbasi* was completely melanized after 1 h (Figure 4d). The capsule was gradually thickened around the nematode and the surface of capsule was fiberizing after 2 h (Figure 4e). The invading nematode was covered with roughly melanized capsule after 4 h (Figure 4f). At 8 h after inoculation, the capsules became heavily melanized around the nematode (Figure 4g). Occasionally, we observed that a few nematodes were partially encapsulated (Figure 4h) and an unencapsulated nematode might form an empty capsule (Figure 4i).

**Figure 5:**
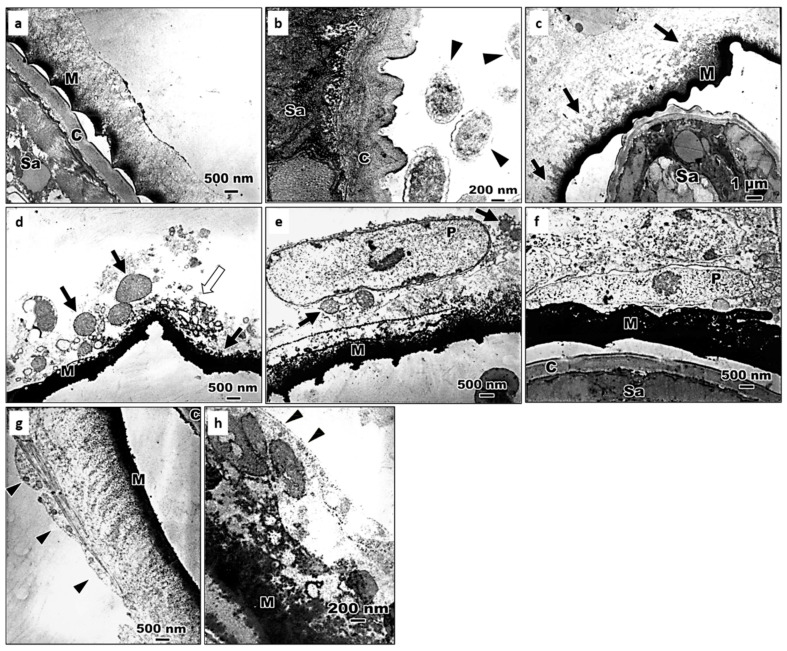
Electron micrographs showing the process of encapsulation of *S. abbasi* in the 4th instar larvae of *Ae. albopictus*. (**a**) At 10 min after inoculation, melanotic capsule deposited on the surface. C: the cuticle of *S. abbasi*; M: melanotic capsule; Sa: *S. abbasi*. (**b**) At 10 min after inoculation, the electron-condense homogeneous material attached to the cuticle of *S. abbasi* and symbiotic bacteria-like structures around *S. abbasi* in the hemocoel of 4th instar larvae. Arrow head (▲): bacteria-like structures; Sa: *S. abbasi*. C: the cuticle of *S. abbasi*. (**c**) At 30 min after inoculation, the inner electron-dense material thickened. Arrows (**↑**): the electron-condense homogeneous materials; M: melanotic capsule; Sa: *S. abbasi*. (**d**) At 1 h after inoculation, cell debris appeared on the out layer of humoral capsule. Black arrows (**↑**): mitochondria; White arrows (**↑**): cell debris; M: melanotic capsule. (**e**) At 1 h after inoculation. M: melanotic capsule; P: plasmatocyte; Arrows (**↑**): mitochondria. (**f**) At 2 h after inoculation, plasmatocyte on the melanotic capsule. C: the cuticle of *S. abbasi*; M: melanotic capsule; P: plasmatocyte; Sa: *S. abbasi*. (**g**) At 24 h after inoculation, intact hemocytes appeared on the humoral capsule. Arrow head: a hemocyte. (**h**) At 48 h after inoculation, the basement membrane-like structures between cellular capsule and hemocoel. Arrow head: the basement membrane-like structures.

Electron micrographs further showed that at 10 min after inoculation of the 4th instar larvae with *S. abbasi*, the homogeneous materials deposited onto the cuticle of *S. abbasi* and electron-dense materials, which appear to be melanin surrounding the surface of *S. abbasi* (Figure 5a) and some symbiotic bacterium-like structures were visualized around *S. abbasi* (Figure 5b). At 30 min after inoculation, the inner electron-dense materials gradually became thickened (Figure 5c). Some cell debris, mitochondria, vacuoles, and intact plasmatocytes attached to the outer surface of the melanotic capsules at 1 h after inoculation (Figure 5d,e). Interdigitated plasmatocytes enclosed the melanotic capsule at 2 h after inoculation (Figure 5f). One layer of plasmatocytes attached to the melanized capsule after 24 h (Figure 5g). Finally, the encapsulation process was complete and the outermost surface of the cellular capsule was surrounded by basement membrane-like materials at 48 h after inoculation (Figure 5h).

## 4. Discussion

Unlike a free-living organism, the IJs of EPNs rely on their host behaviors, body size of different host developmental stages, and host immunity to maximize their chances of a successful infection [36,39,47]. In this study, we demonstrated that this *S. abbasi* isolate exhibited a significant larvicidal activity to 3rd and 4th instar larvae of *Ae. albopictus* in aquatic habitats.

EPNs are often applied as short-term inundative biological control agents in large numbers to bring about a rapid and severe decline in pest numbers [17,23,48], but only a fraction of these successes was found in a host [17]. In addition, the susceptibility of target insects varies depending on nematode species and strains [17,30]. Welch and Bronskill (1962) first reported that *S. carpocapsae* could kill more than 82% of *Ae. aegypti* larvae before pupating although the nematode was shortly encapsulated after penetrating through the gut wall into the hemocoel [46]. They also observed that some EPNs-infected larvae pupated 1 or 2 weeks behind the normal ones, and some failed to pupate and then died after 3 or 4 weeks. Zohdy et al. (2013) indicated that both *S. carpocapsae* and *S. feltiae* failed to establish in larvae of *Culex quinquefasciatus* [38]. This *S. abbasi* isolate could cause a delay in the next molt and pupation (Table 1 and Table S1), but all the nematodes were melanized and encapsulated (Table 2). More recently, steinernematids have been reported to be highly virulent to larvae of *Aedes* spp. [49,50]. Steinernematids are symbiotically associated with *Xenorhabdus* bacteria, which are lethal to many insect species [22]. Tsai et al. (2008) identified the symbiotic bacterium of this *S. abbasi* isolate to be *Xenorhabdus indica* [51]. The mortality rate of *Ae. albopictus* was between 82% and 96% when exposed to *X. indica* for 96 h [52], indicating that the host insects are chiefly killed by symbiotic bacteria. We found that at a concentration of 1000 IJs/mL, IJs of *S. abbasi* and *S. carpocapsae* caused a high mortality of *Ae. albopictus* 3rd and 4th instar larvae, but not the 1st and 2nd instar larvae and pupae. This difference might be due to two reasons: (1) the nematodes are difficult to penetrate directly through the cuticle or per os in the 1st and 2nd instar larvae with a tiny body size [36,37,43,47,53]. (2) The pupal stage does not have natural openings for nematode entry. Edmunds et al. (2017) indicated that, in the aquatic environment, EPNs could survive long enough to parasitize and to kill *Chironomus plumosus* larvae [42]. Steinernematids invade through natural openings and then puncture the gut wall to enter the body cavity in caterpillars and mosquitoes [43,54,55,56]. We also found similar results in *Ae. albopictus* after inoculation with *S. abbasi*. Peters and Ehler (1994) pointed out that an encapsulated *Steinernema feltiae* stuck in the integument of *Tipula oleracea* larva but its posterior end remained outside of the insect body [57]. *Steinernema glaseri* is able to release proteolytic enzymes to assist its penetration through the cuticle [58]. In addition to entry through natural body openings, *S. abbasi* could insert directly into the trumpet, the intersegmental membrane of the cuticle, and the basement of the paddle of *Ae. albopictus* pupae. Although steinernematid species do not have a dorsal tooth or hook on the tip of the head, their invasion can easily accomplish being possibly due to a weak larval cuticle and a lack of exocuticle similar to those of *T. oleracea* [54,57].

Encapsulation is a cellular defense of the insect host against invading parasites that are too large to be phagocytosed. Cellular encapsulation occurs mainly in insects with a large number of hemocytes, for example, lepidopteran species [59,60]. Schmit and Ratcliffe (1977) described cellular encapsulation in *Galleria mellonella* that is formed by the involvement of granular hemocyte and plasmatocytes [61]. However, a cell-free (humoral) encapsulation was induced in *Chinonomus* larvae to combat mermithid nematodes because of a small number of blood cells in Diptera [62,63]. In contrast, Chen and Laurence (1985) reported that the encapsulation of microfilariae in the hemocoel of *Anopheles quadrimaculatus* combined both humoral and cellular encapsulation in which microfilaria was first enclosed in an acellular melanized capsule and then plasmatocytes spread around the humoral capsule to form an outer cellular capsule with one layer of cells [59]. Similarly, the encapsulation of *S. abbasi* in the hemocoel of *Ae. albopictus* larvae combined both humoral and cellular encapsulation in this study (Figure 4 and Figure 5). At 48 h after infection of *An. quadrimaculatus* with a nematode, *Brugia malayi*, the outer surface of the cellular capsule is completely enclosed by basement membrane-like structures, suggesting that these structures laid down to prevent further attachment of any additional hemocytes [64]. We also found that the basement membrane-like structures formed an outer surface of the capsule enclosing *S. abbasi* in the hemocoel of *Ae. albopictus* larvae at 48 h after inoculation (Figure 5h) and the encapsulation structures seem effective in suppressing the development of *S. abbasi*.

Our study showed that at 10 min after inoculation, IJs of *S. abbasi* were observed in the midgut and the gastric caecum of 4th instar larvae, revealing that the IJs could be rapidly ingested by mosquito larvae and effectively killed larvae of *Ae. albopictus*. Notably, after inoculation with EPNs, only few larvae were able to survive to adult emergence (Table 2 and Table S2). It may be due to that the immune systems of mosquitoes overloaded after being exposed to nematode infection, and then the larvae failed to recover and eventually died [65] despite the toxicity of insecticidal proteins produced from the symbiotic bacteria of IJs.

In the present study, high mortalities occurred in the 3rd and 4th instar larvae of *Ae. albopictus*, but not in the 1st and 2nd instar larvae and pupae, indicating that 3rd and 4th instar larvae were more susceptible to *Steinernema* nematodes. Moreover, inoculation of 30 mosquito larvae with *S. abbasi* (1000 IJs/mL) resulted in a higher mortality against 3rd instar larvae than with *S. carpocapsae*.

It was reported that *S. abbasi* is able to kill and produce more IJs in host insects in a temperature ranging from 20 to 30 °C [66,67,68,69,70]. The survival rate of *S. abbasi* was not affected when stored in distilled water up to 6 weeks at 8 °C [71], and even up to 90 days at 30 °C resulting in 70.22% survival [72]. Temperature ranging from 25 to 32 °C was suitable for infectivity and virulence of *S. abbasi* [68]. In our study, approximately 90% larval mortality of *Ae. albopictus* were caused by the infection of this *S. abbasi* isolate within 72 h and were similar to that by *S. carpocapsae* in an aquatic environment. Therefore, this *S. abbasi* isolate when applied to larvae of *Ae. albopictus* can be a potential biocontrol agent for managing this vector mosquito in water.

## 5. Conclusions

In this study, we observed that a *S. abbasi* IJ invaded through the oral route and then punctured the wall of gastric caecum to enter the body cavity of *Ae. albopictus* larvae, and also inserted directly into trumpet, the intersegmental membrane of the cuticle, and the basement of the paddle of pupae. After invading larval hemocoel, the nematodes induced both humoral and cellular encapsulations of *Ae. albopictus* larvae. Whilst this *S. abbasi* isolate could possibly avoid the host cellular defense and cause appreciable mortality of mosquitoes. Therefore, this *S. abbasi* isolate exhibited a significant mosquitocidal effect on the 3rd and 4th instar larvae of *Ae. albopictus* and could thus be considered to be a potential biocontrol agent as an alternative for managing this vector mosquito.

## Figures and Tables

**Table 1:** The time-mortality response of two entomopathogenic nematodes, *Steinernema abbasi* and *S. carpocapsae*, to 3rd- and 4th-instars of *Ae. albopictus* assayed with thirty larvae.

Nematode Species	Mosquito Instars	Lethal Time (LT_50_ and LT_90_) Values (h)			
		1 × 10^3^ (IJs/mL) ^a^		1 × 10^4^ (IJs/mL)	
		LT_50_	LT_90_	LT_50_	LT_90_
*S. abbasi*	3rd	37.9 (35.7–40.1) ^b^	69.1 (67.2–70.9)	31.2 (30.2–32.2)	60.6 (59.7–61.6)
	4th	47.1 (44.6–49.6)	>72 ^c^	34.9 (33.3–36.5)	57.1 (56.1–58.1)
*S. carpocapsae*	3rd	46.4 (45.3–47.4)	>72	31.6 (29.8–33.5)	62.9 (59.8–66)
	4th	47.8 (45.6–49.9)	>72	26.9 (25.7–28.1)	53.1 (51.4–54.8)

^a^ Concentration of nematode suspension. ^b^ The 95% confidence intervals are given in parentheses. ^c^ The mortality rate did not achieve 90% within 72 h.

**Table 2:** The number of encapsulated *Steinernema abbasi* in an *Aedes albopictus* larva and dead/survived mosquito larvae after inoculation.

Number of Encapsulated Nematode in a Mosquito Larva	Number of Mosquito Larvae	
	Dead	Survived
0	0	7
1	25	6
2	47	4
3	28	0
4	25	1
5	10	0
6	9	0
7	7	0
8	3	0
9	2	0
10	1	0
11	1	0
13	1	0
14	1	0
16	1	0
17	1	0
20	1	0
31	1	0
**Total**	164	18
**Percentage (%)**	90.11	9.89

## Supplementary Materials

The following are available online at https://www.mdpi.com/2075-4450/11/12/832/s1. Figure S1: The mortality of *Aedes albopictus* larvae inoculated with *Steinernema abbasi* or *S. carpocapsae* at concentrations of 0, 1, 10, 100, 1000, and 10,000 IJs/mL at 72 h after inoculation; Table S1: The development time (mean ± SD) of immature stages of *Aedes albopictus* at 28 ± 1 °C; Table S2: The number of encapsulated *Steinernema carpocapsae* and degree of encapsulation in *Aedes albopictus* larvae.
