# Supplementary material for: The Invasion and Encapsulation of the Entomopathogenic Nematode, Steinernema abbasi, in Aedes albopictus (Diptera: Culicidae) Larvae
Source: Insects. 2020 Nov 26;11(12):832. doi: 10.3390/insects11120832 (PMC7760258; doi:10.3390/insects11120832)
Supplement: Supplementary file 1 [file insects-11-00832-s001.pdf]

Supplementary files

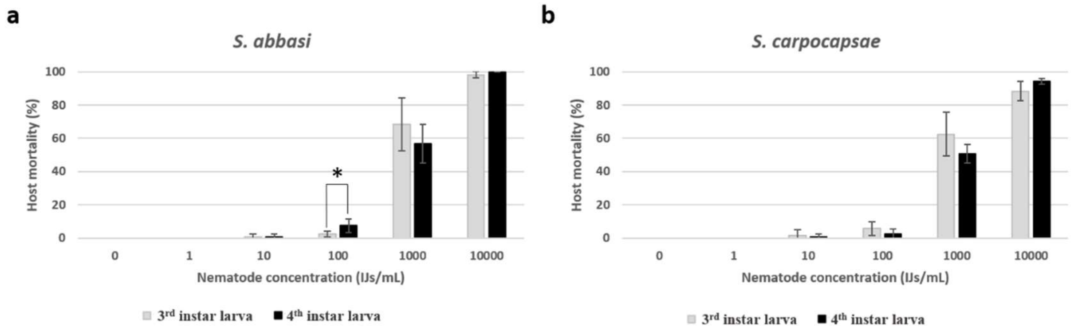

Figure S1. The mortality of *Aedes albopictus* larvae inoculated with *Steinernema abbasi* or *S. carpocapsae* at concentrations of 0, 1, 10, 100, 1,000 and 10,000 IJs/ml at 72 h after inoculation. Asterisks indicate a significant difference in the mortality of 4th instar larvae as compared to the mortality of 3rd instar larvae in series of increasing nematode concentrations (One-way ANOVA; Tukey's honestly significant difference (HSD) test; $P < 0.05$). (a-b) Thirty *Ae. albopictus* in 30 ml of water.

Table S1. The development time (mean ± SD) of immature stages of *Aedes albopictus* at 28 ± 1°C

Larval stages (h)				Pupal stage
1st instar	2nd instar	3rd instar	4th instar	
25.9 ± 1.04	33.1 ± 0.39	42.4 ± 0.71	71.02 ± 1.98	54.1 ± 2.07

Table S2. The number of encapsulated *Steinernema carpocapsae* in an *Aedes albopictus* larva and dead/survived mosquito larvae after inoculation

Number of encapsulated nematode in a mosquito larva	Number of mosquito larvae	
	**Dead**	**Survived**
0	0	4
1	48	0
2	58	2
3	35	0
4	18	0
5	4	0
6	4	0
7	2	0
11	1	0
15	1	0
**Total**	171	6
**Percentage (%)**	96.6	3.4
